# Initial experience of anti-PD1 therapy with nivolumab in advanced hepatocellular carcinoma

**DOI:** 10.18632/oncotarget.20029

**Published:** 2017-08-08

**Authors:** Duan Feng, Xie Hui, Lu Shi-Chun, Bai Yan-Hua, Cui Li, Li Xiao-Hui, Yan Jie-Yu

**Affiliations:** ^1^ Department of Interventional Radiology, The General Hospital of Chinese People’s Liberation Army, Beijing, China; ^2^ Department of Interventional Therapy, 302 Hospital of Chinese People’s Liberation Army, Beijing, China; ^3^ Department of Hepatobiliary Surgery, The General Hospital of Chinese People’s Liberation Army, Beijing, China

**Keywords:** hepatocellular carcinoma, nivolumab, anti-PD1, immunotherapy, Immunology and Microbiology Section, Immune response, Immunity

## Abstract

**Purpose:**

To evaluate efficacy and safety of anti-PD1 therapy with nivolumab for treatment of advanced hepatocellular carcinoma (HCC).

**Methods:**

From Jan 2016 to Jan 2017, eleven cases of HCC (average age of 51.8-year), 4 at stage B and 7 at stage C, according to Barcelona Clinic Liver Cancer staging, were treated with nivolumab. There were 4 patients with lung metastasis, 1 with portal vein tumor thrombus, 1 with abdominal metastasis and 1 with bone metastasis. The protocol was nivolumab, 3 mg/kg, on day 1, q3w. All patients were treated for more than 4 cycles. During anti-PD1 treatment period, 6 patients also received sorafenib and 1 patient received cytokine-induced killer cell therapy. Objective response and clinical adverse events were evaluated retrospectively.

**Results:**

Patients underwent a total of 80 cycles of nivolumab therapy, ranging between 4 and 18 cycles per patient. Nivolumab was associated with a disease control rate of 81.8%, with an objective response of 63.6% (Modified Response Evaluation Criteria in Solid Tumors). No adverse effects related to nivolumab were noted.

**Conclusion:**

Our experience shows that nivolumab could achieve acceptable outcome in HCC patients and may serve as an optional treatment, especially for patients who failed to gain a benefit from routine treatments.

## INTRODUCTION

Hepatocellular carcinoma (HCC) is one of the most common malignant tumors and the second leading cause of cancer-related death worldwide [1, 2]. Currently, surgery and minimal invasive treatments (for example, ablation, trans-arterial chemoembolization) are main options for the treatment of HCC [3]. However, for advanced HCC, whole body treatment is unavoidable. Up to now, sorafenib is the only molecular targeted drug that can effectively prolong lifespan in HCC patients, which has been approved by the American FDA. Unfortunately, sorafenib prolongs survival on average of only 3 months and the response rate is low [4, 5]. Therefore, it is of critical importance to develop new and more effective treatment protocols for advanced HCC.

Manipulation of immune checkpoints such as programmed death-1 (PD1) with targeted antibodies has recently emerged as an effective anticancer strategy [6]. Nivolumab, as a novel immune checkpoint inhibitor, is increasingly used in patients with cancer, primarily melanoma, and is currently under evaluation for a number of other cancers [7]. As Nivolumab shows great potential, we evaluated efficacy and safety of this approach in 11 HCC patients with advanced disease.

## RESULTS

**Figure 1:**
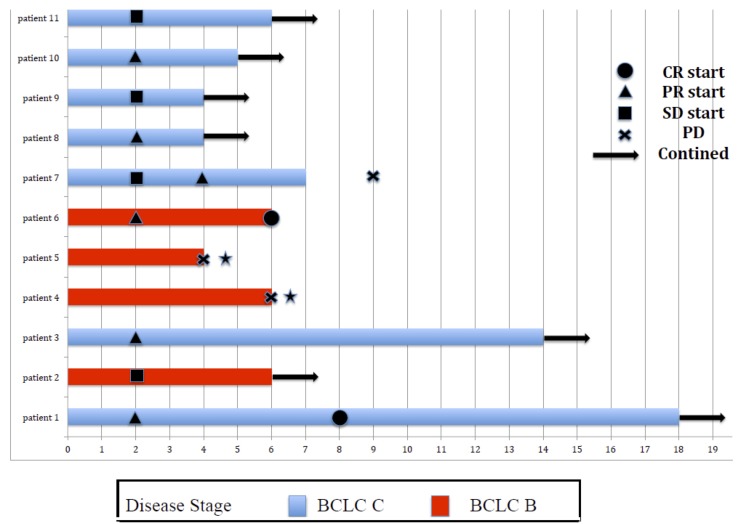
Swimmer plot of 11 patients who received at least 4 cycles of nivolumab *Patient died.

**Figure 2:**
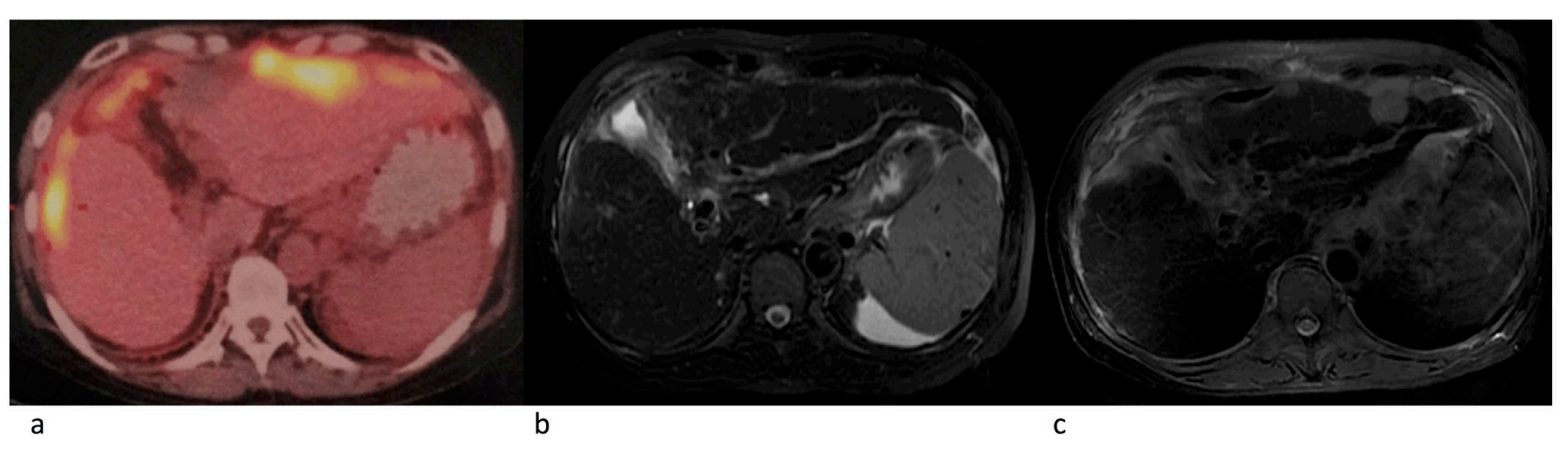
Radiographies of a 54-year old female patient PET-CT showed multiple lesions in bilobar, MRI (T2, after 7 cycles with nivolumab) showed lesions significant decreased. MRI (T2, two months after nivolumab was discontinued) showed progressive disease. **a.** Before nivolumab treatment. **b.** Seven cycles after nivolumab treatment. **c.** Two months later after nivolumab withdrawal.

**Figure 3:**
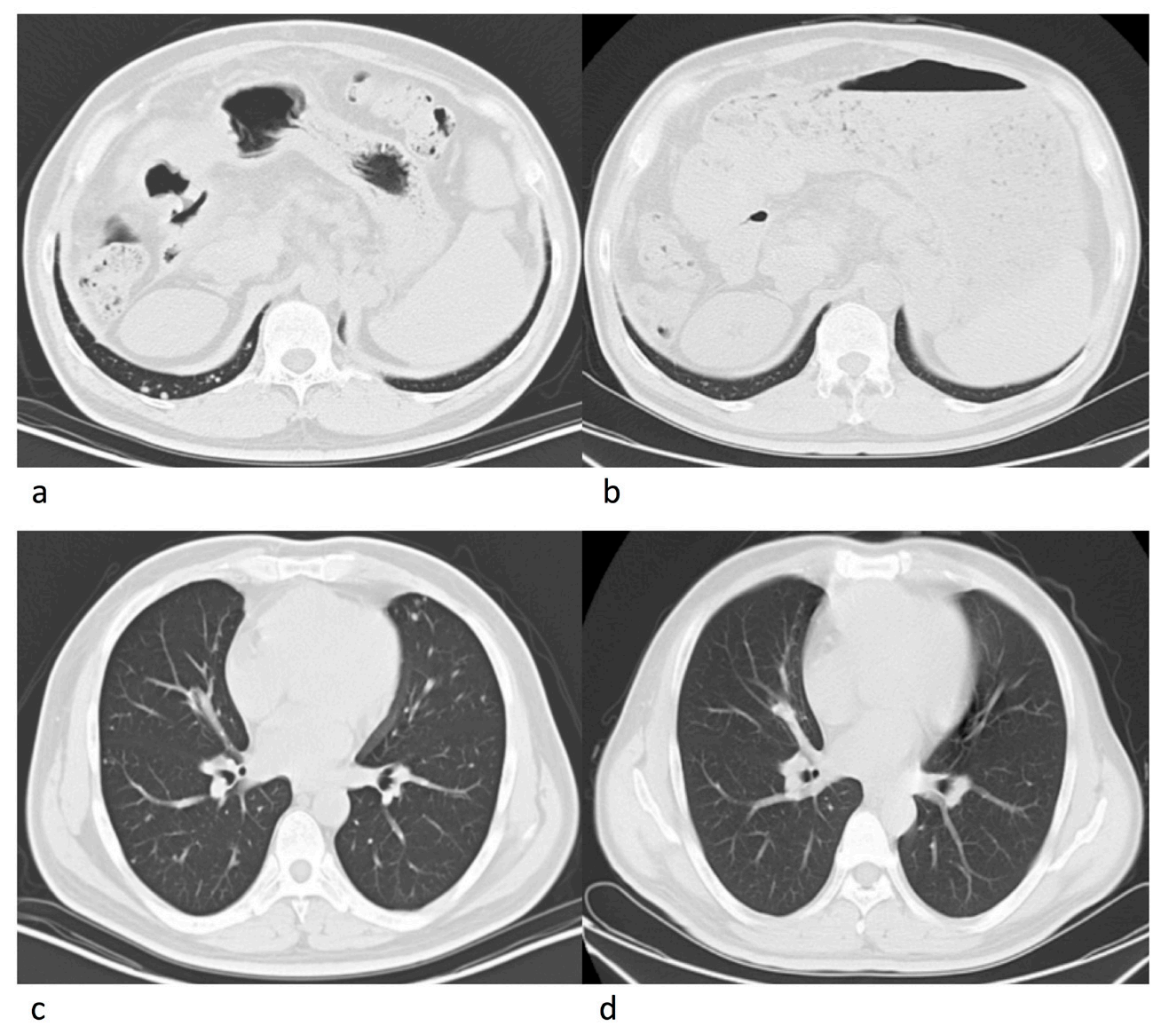
Radiographies of a 43-year old male patient Tumors in lungs disappeared after 8 cycles of nivolumab. **a.**, **c.** Pre-treatment. **b.**, **d.** Post-treatment.

**Figure 4:**
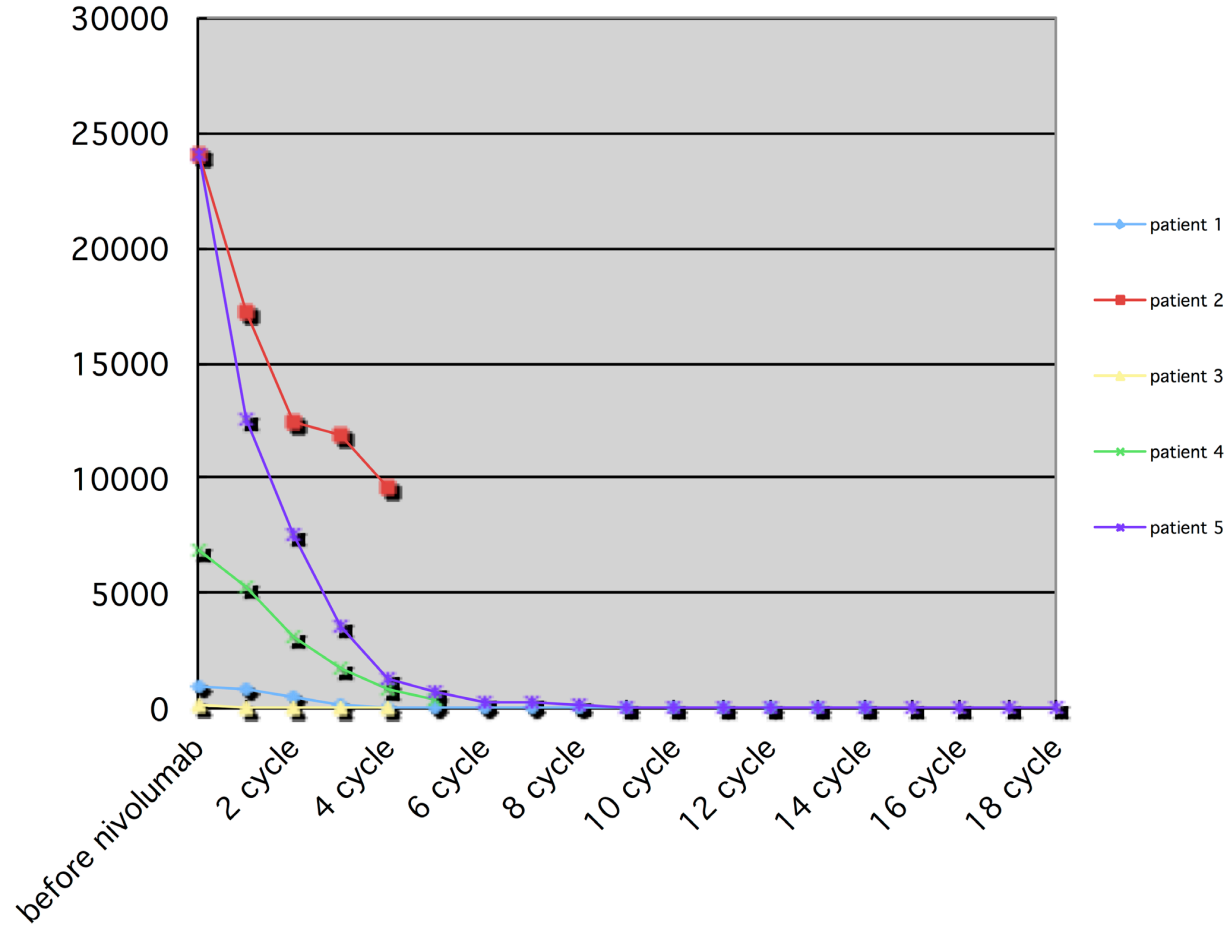
Trend of AFP after nivolumab treatment, in 5 patients who previously had elevated AFP

Eleven patients were eligible for response evaluation (Figure 1). One patient previously had resection of multiple intrahepatic tumors and thoracic vertebrae metastasis (nivolumab only, 18 cycles), and one patient had multiple intrahepatic tumors with no distant metastasis after TACE treatment (nivolumab combined with CIK cell therapy, 6 cycles), during the treatment, both patients had complete response (CR). Five patients had partial response (PR), one of which stopped treatment after the majority of intrahepatic tumors were under control, thus experienced tumor progression 2 months later (Figure 2). One had recurrence in the liver after disappearance of lung metastasis, and underwent one round of TACE in addition to the scheduled treatment (Figure 3). Two patients had stable disease (SD), one had a thrombus in the right branch of the portal vein, and the other patient had lung metastases. Two patients had progressive disease (PD), both had multiple intrahepatic tumors and later died of liver failure during follow-up (after 4^th^ and 6^th^nivolumab treatment cycle). Five patients had elevated alpha-fetoprotein (AFP) before treatment started, while AFP values in all these patients decreased after nivolumab treatment (Figure 4).

None of the patients developed metastasis other than at their existing distant metastasis locations. Overall, nivolumab was associated with a disease control rate of 81.8%, with an objective response of 63.6%.

One patient developed hemorrhage of the digestive tract after 6 cycles of treatment, which was considered to be caused by portal hypertension, resulting in esophagogastric varicose hemorrhage. Treatment was terminated immediately, and bleeding was stopped after symptomatic treatment. For the two patients who died of liver failure, we consider tumor progression as the cause of death. No other adverse effects were observed.

## DISCUSSION

After decades of research, immune therapy for cancer treatment has eventually progressed from bench to bed, considerably resulting from research on immune checkpoint inhibitors [8]. Anti-PD1 therapy demonstrated promising results against multiple tumor types including: melanoma, non-small cell lung cancer (NSCLC) [9, 10], and head and neck squamous cell carcinoma [11]. Nivolumab is a full human IgG4 anti-PD1 mAb, which has been approved by FDA for patients with advanced melanoma in 2014, and for patients with metastatic NSCLC in 2015 [12, 13]. And the combination of ipilimumab and nivolumab was recently approved in the patients with advance melanoma in Septembe, 2015 [14]. In checkmate 040 study [15], patients received intravenous nivolumab 0.1mg/kg, 0.3mg/kg, 1mg/kg, 3mg/kg and 10 mg/kg in different groups, every 2 weeks. Nivolumab showed manageable safety and durable objective responses in both dose escalation cohort and dose expansion cohort. Our protocol was 3mg/kg, every 3 weeks and our retrospective analysis showed potential clinical benefit (less toxicity than checkmate 040) of nivolumab in patients with advanced HCC.

The genetic test for PD1 and PDL1 prior to anti-PD1 antibody was not performed in our study, although it was reported this test prior anti-PD1 antibody treatment improves prediction of treatment efficacy [16]. The following concerns were raised for the absence of genetic test. All patients were hepatitis B virus (HBV) positive. According to literature [17], microenvironment in the liver of HBV patients can promote T-cell exhaustion, which is often accompanied by high-level expression of PD1 and PDL1. It has been shown that the use of anti-PD1 antibody can even efficiently suppress HBV infection [18,19]. Moreover, it is important to note that not all patients with high level of PD1 expression are sensitive to nivolumab, for instance, patients with a hepatitis C virus (HCV) infection, besides high level of PD1 expression, HCV patients have also high level expression of CTLA-4 and low level expression of CD28 and CD127 [20]. An *ex vivo* study indicates that dual CTLA-4 and PD1 blockade, but not individual blockade, is required to reverse HCV specific effector T-cell dysfunction [21]. Therefore, for HCV-derived HCC, immune checkpoint inhibitor monotherapy may have limited effects.

Immune checkpoint blockade for HCC is currently being evaluated in combination with other treatments including TACE and ablation. However, none of the published studies on these combinations are based on a randomized controlled treatment protocol. Evidence on combinational therapy involving nivolumab is still lacking. It remains a question that which treatment method can be combined with nivolumab to achieve reliable and superior efficacy remains a question [22]. Similarly, although satisfying effects (4 PR, 1 SD, 1 PD) were achieved in the patients who received sorafenib or cell therapy without treatment related complizaiotns in our study, our retrospective study indicates benefit of nivolumab in combination with sorafenib, which needs additional studies to provide evidence. Nevertheless, based on the positive results from this study, application of nivolumab combined with other therapies should be further explored.

Although immune-checkpoint blocking antibodies are effective in many types of cancers, many immune mediated adverse events should not be neglected. Besides common side-effects, such as atypical pneumonia, colitis, hepatitis, rash and endocrinopathies [23, 24]. There are also uncommon side-effects, such as cardiotoxicity, vision and hearing loss, and lupus nephritis [25, 26, 27]. No nivolumab-related side effects were observed in this study. Additionally, according to our knowledge, side effects related to nivolumab in liver cancer treatment have not been reported thus far. Our results indicate that nivolumab might be a relatively safe drug for the treatment of liver cancer.

It has been controversial for the end point that stops the nivolumab treatment. In patient #7 (Figure 1), tumor progression was observed after stop intaking nivolumab when complete tumor suppression was achieved after 7 cycles of nivolumab treatment. In patient #1 (Figure 1), 10 more cycles was continued when AFP decreased to normal range and no viable tumors were detected on whole-body imaging after 8 cycles of therapy. In the imaging following up, this patient has been estimated as CR. Based on these two cases, we like to suggest that it is not recommended to stop nivolumab prematurely, after an initial therapeutic effect is achieved. However, it is not clear from our study whether prolongation of treatment would further improve clinical outcome. It has been reported that long-term application of anti-PD1 antibody can also cause drug resistance [28]. Thus, the duration of nivolumab application for HCC treatment needs to be further studied.

Here we show safety and beneficial outcome of anti-PD1 therapy with nivolumab in advanced HCC patients. The study has several limitations including the retrospective nature and limited number of patients etc. However, our results warrant further study on combination therapy with nivolumab in advanced HCC.

## MATERIALS AND METHODS

### Patients and treatment protocol

Between Jan 2016 and Jan 2017, a total of 11 patients who were diagnosed with advanced HCC were treated with nivolumab. Baseline patient characteristics are summarized in Table 1. Four patients in Barcelona-Clinic Liver Cancer (BCLC) stage B and seven patients in BCLC stage C. Four patients with lung metastasis, two patients with portal vein (right branch) tumor thrombus and one patient with bone metastasis. Three patients underwent hepatectomy previously, 1 received radiation therapy, 6 received sorafenib, and all patients underwent 1-5 sessions of trans-arterial chemoemobolization (TACE). This study was approved by the institutional review board (IRB) of Chinese PLA General Hospital.

**Table 1:** Patient baseline characteristics

Factor	
**Age**	
**Median**	54.8
**Range**	42-70
**Sex**	
**Male**	8
**Female**	3
**Etiology**	
**HBV**	11
**BCLC stage**	
**B**	4
**C**	7
**AFP value**	
**Normal range (0-20 ug/L)**	6
**Higher than normal range**	5
**ECOG PS**	
**0**	9
**1**	2
**Nivolumab**	
**Cycles (median)**	7.1
**Range**	4-18

Three days before each cycle of nivolumab treatment, the patients were tested for blood cell count, liver and renal panels and serum tumor markers. Baseline imaging included plain computed tomography (CT) scans of the chest, tri-phase CT scan or dynamic magnetic resonance imaging (MRI) of the abdomen. Fluorodeoxyglucose positron emission tomography (PET) scan was optional. Imaging was performed every other cycle. Treatment commenced when following criteria were met: WBC ≥ 4.0*10^9^, platelets ≥ 50*10^12^, RBC ≥ 3.5*10^9^, liver function Child-Pugh score < 8, and kidney function within normal range. Nivolumab was dosed at 3 mg/kg IV every 3 weeks. All patients were treated for more than 4 cycles. During nivolumab treatment, 6 patients also continued with their previous treatment of sorafenib, and 1 patient also received cytokine-induced killer (CIK) cell therapy. The protocol for this patient was nivolumab treatment on day 1, CIK cell therapy on day 2 and 3, consisting of a singular cell transfusion of > 8*10^9^.

### Evaluation

Responses were defined using Modified Response Evaluation Criteria in Solid Tumors criteria [29] based on an enhanced abdominal CT or MRI. Side effects and toxicity related to the treatment were graded according to NCI-CTCAE version 4.0 [30].
